# Diaphragm function does not independently predict exercise intolerance in patients with precapillary pulmonary hypertension after adjustment for right ventricular function

**DOI:** 10.1042/BSR20190392

**Published:** 2019-09-03

**Authors:** Jens Spiesshoefer, Simon Herkenrath, Michael Mohr, Winfired Randerath, Izabela Tuleta, Gerhard Paul Diller, Michele Emdin, Peter Young, Carolin Henke, Anca Rezeda Florian, Ali Yilmaz, Matthias Boentert, Alberto Giannoni

**Affiliations:** 1 Respiratory Physiology Laboratory, Department of Neurology with Institute for Translational Neurology, University Hospital Muenster, Muenster, Germany; 2 Bethanien Hospital GmbH Solingen, Solingen, Germany and Institute for Pneumology at the University of Cologne, Solingen, Germany; 3 Department of Medicine A, Hematology, Oncology and Pulmonary Medicine, University Hospital Muenster, Muenster, Germany; 4 Department of Cardiology I, University Hospital Muenster, Muenster, Germany; 5 Department of Cardiology III, University Hospital Muenster, Muenster, Germany; 6 Cardiology and Cardiovascular Medicine Division, Fondazione Toscana Gabriele Monasterio, CNR-Regione Toscana, Pisa, Italy; 7 Institute of Life Sciences, Scuola Superiore Sant’Anna, Pisa, Italy; 8 Department of Neurology, Medical Park Klinik Reithofpark, Bad Feilnbach, Germany

**Keywords:** diaphragm function, exercise tolerance, pulmonary hypertension, right heart failure

## Abstract

**Background:** Several determinants of exercise intolerance in patients with precapillary pulmonary hypertension (PH) due to pulmonary arterial hypertension and/or chronic thromboembolic PH (CTEPH) have been suggested, including diaphragm dysfunction. However, these have rarely been evaluated in a multimodal manner. **Methods:** Forty-three patients with PH (age 58 ± 17 years, 30% male) and 43 age- and gender-matched controls (age 54 ± 13 years, 30% male) underwent diaphragm function (excursion and thickening) assessment by ultrasound, standard spirometry, arterial blood gas analysis, echocardiographic assessment of pulmonary artery pressure (PAP), assay of amino-terminal pro-brain natriuretic peptide (NT-proBNP) levels, and cardiac magnetic resonance (CMR) imaging to evaluate right ventricular systolic ejection fraction (RVEF). Exercise capacity was determined using the 6-min walk distance (6MWD). **Results:** Excursion velocity during a sniff maneuver (SniffV, 4.5 ± 1.7 vs. 6.8 ± 2.3 cm/s, *P*<0.01) and diaphragm thickening ratio (DTR, 1.7 ± 0.5 vs. 2.8 ± 0.8, *P*<0.01) were significantly lower in PH patients versus controls. PH patients with worse exercise tolerance (6MWD <377 vs. ≥377 m) were characterized by worse SniffV, worse DTR, and higher NT-pro-BNP levels as well as by lower arterial carbon dioxide levels and RVEF, which were all univariate predictors of exercise limitation. On multivariate analysis, the only independent predictors of exercise limitation were RVEF (r = 0.47, *P*=0.001) and NT-proBNP (r = −0.27, *P*=0.047). **Conclusion:** Patients with PH showed diaphragm dysfunction, especially as exercise intolerance progressed. However, diaphragm dysfunction does not independently contribute to exercise intolerance, beyond what can be explained from right heart failure.

## Introduction

Pulmonary arterial hypertension (PAH) and chronic thromboembolic pulmonary hypertension (CTEPH) as forms of precapillary pulmonary hypertension (PH) are associated with significant exercise intolerance, but the exact mechanisms leading to exercise limitation are multifactorial and still not well understood [1–6].

In precapillary PH, and in PAH and CTEPH in particular, increased pressure in the pulmonary arteries overloads the right ventricle (RV), causing hypertrophy and failure [5]. The majority of measures that predict survival in PH, such as exercise capacity and functional class, have been related to RV function [5]. In particular, RV function was shown to be the only independent predictor of exercise intolerance when evaluated together with systolic pulmonary artery pressure (PAP) [6,7].

Recently, inspiratory muscle strength has emerged as a potential new contributor to exercise intolerance in PH [8,9]. Indeed, lower values of inspiratory muscle strength as assessed by transdiaphragmatic pressure following magnetic stimulation of the phrenic nerve roots were shown to relate to exercise intolerance in patients with precapillary PH [8]. While this showed that inspiratory muscle dysfunction parallels the development of exercise intolerance in PH, it is not yet known whether the diaphragm contribution to exercise intolerance is independent of RV systolic dysfunction in precapillary PH.

Therefore, this prospective study was conducted in patients with precapillary PH and age- and sex-matched controls to evaluate whether diaphragm function, assessed by diaphragm ultrasound, [10] contributes to exercise intolerance, evaluated by the 6-min walking test, over and above impaired right ventricular pump function, as assessed by cardiac magnetic resonance (CMR) imaging.

## Experimental

### Study design

The present study was conducted at the University Hospital of Münster (Universitätsklinikum Münster) from June to September 2018. Informed consent was obtained from each subject and the study protocol conformed to the ethical guidelines of the 1975 Declaration of Helsinki as reflected in *a priori* approval by the Institution’s Human Research Committee (Ethikkommission der Ärtzekammer Westfalen Lippe, ethical approval number 2017-187-f-S). All participants gave verbal written informed consent to participate in the study. The project was prospectively registered and continuously updated under the German Clinical Trials Registry (drks.de Identifier: DRKS00014695).

### Study population

Patients with Nizza class I PAH or Nizza class IV CTEPH were consecutively recruited based on the latest European Society of Cardiology (ESC) criteria [1]. All patients underwent the following tests as per the latest recommendations and guidelines: standard two-dimensional echocardiography for estimating PAP; [1] CMR imaging to quantify right ventricular function; [11] spirometry to assess lung function; [12,13] capillary blood gas analysis; diaphragm ultrasound; and 6-min walking test as a measure of exercise capacity.

A group of healthy volunteers well matched for age, gender and body mass index (BMI) was enrolled as control group and underwent evaluation of diaphragm function. All volunteers were required to have normal electrocardiogram (ECG) findings, pulmonary function natriuretic peptide hormone levels.

### CMR data acquisition and analysis

ECG-gated CMR studies were performed on a 1.5-T scanner (Ingenia, Philips, Best, The Netherlands) using commercially available cardiac software and cardiac-dedicated surface coils. Cine-imaging was performed using a steady-state-free-precession (SSFP) sequence in four long-axis slices (four-, three, two-chamber as well as a modified two-chamber for the RV) and a stack of short-axis slices completely covering the ventricles.

CMR analysis was performed off-line by two experienced readers. Ventricular volumes and ejection fraction were derived by contouring the endocardial borders at end-diastole and end-systole on the short-axis cine images and including the left ventricular (LV) papillary muscles/RV trabeculations in the ventricular cavity. For optimal segmentation of the RV, the tricuspid valve and apical planes were first defined using the four- and modified two-chamber for RV slices. RV systolic dysfunction was defined according to the latest available published data [11].

### Diaphragm ultrasound

A portable ultrasound machine (LOGIQ S8 -XD clear, GE Healthcare, London, United Kingdom) with a 3.5-MHz convex transducer was used for assessment of diaphragm excursions in the subcostal view, and a 10-MHz linear transducer was used for evaluation of diaphragm thickness in the zone of apposition. Measurements were performed on the right hemidiaphragm in the supine position because posture is known to directly affect diaphragm thickness [14]. All measurements were performed three times and the average value for each parameter was then calculated.

**Figure 1:**
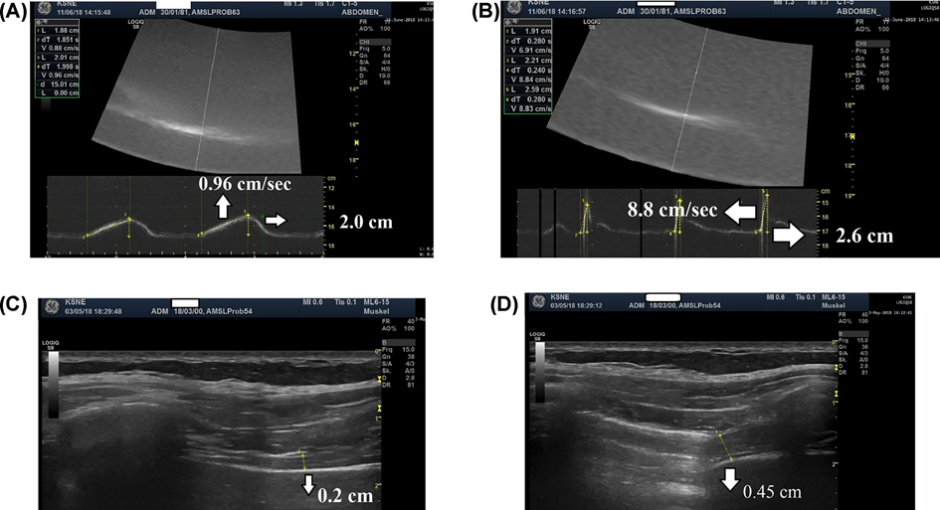
Standardised Diaphragm Ultrasound Protocol Parameters measured during diaphragm ultrasound: diaphragm excursion during TB (**A**) and sniff maneuver (**B**); and diaphragm thickness at FRC (**C**) and TLC (**D**).

For evaluation of diaphragm excursions, the 3.5 MHz probe was positioned between the mid-clavicular and anterior axillary lines, in the subcostal area with the probe held as medially as possible and directed cranially (Figure 1A). Excursions of the right hemidiaphragm were recorded on M-Mode sonography in real time with a clear instruction to ‘cut’ the hemidiaphragm in its posterior third. Measurement of diaphragm excursion amplitude was performed during tidal breathing (TB) (Figure 1A), after deep inspiration toward total lung capacity (TLC) and following a voluntary sniff (VS) maneuver which leads to maximum displacement of the diaphragm. Assessment of diaphragm excursion velocity was performed during TB and following the VS maneuver only (Figure 1B). Excursion amplitude was defined as the upright-perpendicular distance from the minimum to the maximum point of diaphragm displacement, and excursion velocity was defined as the upright-diagonal distance from the minimum to the maximum point. Diaphragm thickness was measured as the vertical distance between the pleural and peritoneal layer at both TLC and functional residual capacity (FRC) (Figure 1B). This was done in the zone of apposition, which is defined as the chest wall area where the abdominal contents reach the lower ribcage. In a standardized manner, we first visualized the diaphragm with the 10 MHz probe positioned in the posterior axillary line between the eighth and tenth intercostal space. Thus, the diaphragm can be easily visualized by its characteristic three layers (pleural and peritoneal layer as echogenic outer borders; Figure 1C). During inspiration, the diaphragm contracts and gets thicker, and on expiration it relaxes getting thinner again (Figure 1C,D). Diaphragm thickness was defined as the distance from the inner part of the pleural layer to the inner part of the peritoneal layer, measured at its thickest portion adjacent to the lung. Diaphragm thickening ratio (DTR) was calculated as thickness at TLC divided by thickness at FRC.

### Statistical analysis

All analyses were performed using Sigma Plot™ software (Version 13.0, Systat Software Ltd, Erkrath, Germany). Before analysis, data distribution was tested using the Kolmogorov–Smirnov test. Results are expressed as mean and standard deviation or median and interquartile ranges for continuous variables with normal or skewed distribution, respectively, and as percentages for categorical data. Comparison of continuous variables between groups was done using independent *t* test or Mann–Whitney U test, as appropriate, while categorical data were compared using the Chi-Square test, with Fisher’s exact test, as appropriate. Pearson product moment correlation was used to explore the relationship between continuous variables: strength of correlation was classified as weak (0.20–0.38), moderate (0.39–0.58), strong (0.58–0.78) or very strong (0.79–1.00). Determinants of exercise intolerance were evaluated using univariate and multivariate regression analyses forcing age and gender into the equation with 6-min walk distance (6MWD) as the dependent variable. Based on pilot data and assuming a two-sided significance level of 5% (α) and 80% power (β), a sample size of 35 patients was calculated to be required to detect a 20% reduction in DTR. To allow for dropouts, the target sample size was 43 patients and the same number of controls. For all analyses, a *P*-value of <0.05 was considered statistically significant.

## Results

### Subjects

Forty-three consecutive patients with precapillary PH (age 58 ± 17 years, 30% male, BMI 28 ± 7 kg/m^2^) and 43 control subjects matched for gender, age, and BMI (age 54 ± 13 years, 30% male, BMI 29 ± 8 kg/m^2^) were enrolled in the study (Table 1). The majority of patients with precapillary PH had PAH (*n*=36) and the others had CTEPH (*n*=7).

**Table 1:** Demographic and clinical characteristics of the study population at baseline

	Patients with precapillary PH (*n*=43)	Healthy volunteers (*n*=43)
Male, *n* (%)	13 (30)	13 (30)
Age, years	57.8 ± 17.1	53.5 ± 12.6
BMI, kg/m^2^	27.6 ± 6.7	28.9 ± 7.8
BSA, m^2^	2.0 ± 0.2	2.0 ± 0.2
NYHA class, *n* (%)		
I	1 (2)	-
II	16 (37)	-
III	25 (58)	-
IV	1 (2)	-
Systolic PAP, mmHg	51 ± 7	-
RVEF, %	50.1 ± 12.3	-
Impaired RVEF, *n* (%)	27 (63)	-
NT-proBNP, pg/ml	860 (200–1159)	<50
Capillary CO_2_, mmHg	33.4 ± 4.3	-
**Lung function data**		
FVC, L	2.62 ± 1.0	4.5 ± 1.1^1^
FVC, % predicted	76.0 ± 18.2	106 ± 15^1^
FEV_1_/VC, %	77 ± 10	81 ± 8
**Medication, *n* (%)**		
PDE 5-I	20 (49)	-
Dir.cGMP Stim	5 (12)	-
ERA	25 (61)	-
PCA	6 (15)	-

Values are mean ± standard deviation, median (interquartile range) or number of patients (%). Abbreviations: BSA, body surface area; Capillary CO_2_, capillary carbon dioxide level; Dir.cGMP Stim, direct cyclic guanosine monophosphate stimulator; ERA, endothelin receptor antagonist; FEV_1_, forced expiratory volume after 1 s; FVC, forced vital capacity; NT-proBNP, amino-terminal pro-brain natriuretic peptide; NYHA, New York Heart Association; PCA, prostacyclin analog; PDE 5-I, phosphodiesterase 5 inhibitor; RVEF, right ventricular ejection fraction.

1 *P*<0.05 vs patients with precapillary PH.

PH patients had moderate to severe PH (systolic PAP 51 ± 7 mmHg) as indicated by the fact that most (58%) were in New York Heart Association (NYHA) functional class III with moderate to severe exercise impairment (based on 6MWD achieved). Patients with PH also had systolic RV dysfunction (63% of cases) and elevated NT-pro-BNP levels. Respiratory data revealed a mild but significant reduction in forced vital capacity (FVC) and the presence of hypocapnia (capillary carbon dioxide) CO_2_] <35 mmHg in 71%).

### Diaphragm ultrasound measures

During TB, the diaphragm moved significantly faster and wider in precapillary PH patients compared with controls (diaphragm width: 2.5 ± 1.8 vs 1.6 ± 0.5 cm, *P*<0.01; diaphragm velocity: 1.7 ± 0.9 vs. 1.1 ± 0.4 cm/s, *P*<0.01). Excursion velocity during a sniff maneuver (SniffV, 4.5 ± 1.7 vs. 6.8 ± 2.3 cm/s, *P*<0.01) and the DTR (1.7 ± 0.5 vs. 2.8 ± 0.8, *P*<0.01) were significantly reduced in precapillary PH patients versus controls (Table 2). Considering the lower limit of normality (95% confidence interval) obtained in the healthy population (6.11 cm/s for SniffV and 2.49 for DTR), precapillary PH patients showed impaired SniffV and DTR in 91 and 84% of cases, respectively.

**Table 2:** Diaphragm ultrasound measures in patients with precapillary PH and healthy volunteers

	Patients with precapillary PH (*n*=43)	Healthy volunteers (*n*=43)	*P*-value
**Diaphragm excursion**			
Amplitude during TB, cm	2.5 ± 1.8	1.6 ± 0.5	**0.003**
Velocity during TB, cm/s	1.7 ± 0.9	1.1 ± 0.4	**<0.001**
Amplitude during VS, cm	2.9 ± 1.0	2.6 ± 1.1	0.116
Velocity during VS, cm/s	4.5 ± 1.7	6.8 ± 2.3	**<0.001**
Amplitude during maximal inspiration, cm	5.0 ± 1.3	7.8 ± 1.9	**<0.001**
**Diaphragm thickness**			
FRC, cm	0.23 ± 0.09	0.20 ± 0.07	0.309
TLC, cm	0.37 ± 0.11	0.53 ± 0.19	**<0.001**
Thickening ratio	1.7 ± 0.5	2.8 ± 0.8	**<0.001**

Values are mean ± standard deviation.

### Clinical and instrumental characteristics of precapillary PH patients with exercise intolerance

**Figure 2:**
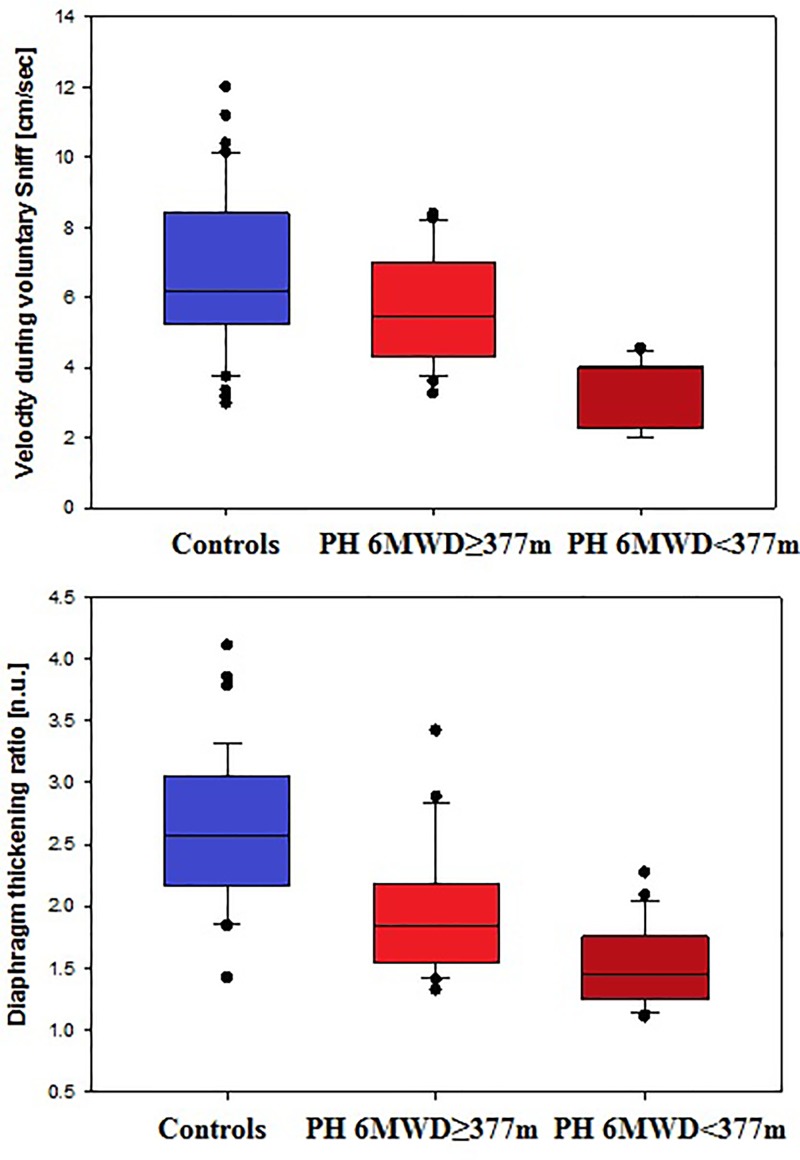
Diaphragm velocity during a VS maneuver (**top**) and DTR (**bottom**) in controls (left blue box plot) versus patients with precapillary PH and less (6WMD ≥377 m) (mid box light red plot) or more (6MWD <377 m) (right dark red box plot) pronounced exercise intolerance (all *P*<0.01; see Tables 1 and 2)

When dichotomized into two groups based on 6MWD above or below the median, precapillary PH patients with more pronounced exercise intolerance (6MWD <377 vs. ≥377 m) had worse right ventricular systolic ejection fraction (RVEF) (45 ± 13 vs. 55 ± 10%, *P*<0.01), higher levels of NT-pro-BNP (1000 [interquartile ratio (IQR): 557–2689] vs. 450 [IQR: 109–1000] ng/l, *P*<0.01) and lower pCO_2_ values (31 ± 4 vs. 35 ± 4 mmHg, *P*<0.01). They also showed worse SniffV (3.4 ± 1.0 vs. 5.7 ± 1.5 cm/s, *P*<0.01) and worse DTR (1.5 ± 0.3 vs. 2.0 ± 0.6, *P*<0.01) (Table 3 and Figure 2). Of note, no significant between-group differences were seen in systolic PAP, FVC, or forced expiratory volume after 1 s (FEV_1_). Supplemental Videos S1 and S2 summarize diaphragm ultrasound abnormalities found in a patient with preserved RV systolic function and in a patient with impaired RV systolic function.

**Table 3:** Characteristics of precapillary PH patients dichotomized by 6MWD

	Patients with precapillary PH		*P*-value
	6MWD <377 m (*n*=21)	6MWD ≥377 m (*n*=22)	
Age, years	64.5 ± 14.5	51.5 ± 17.1	**0.012**
Male, *n* (%)	4 (19)	9 (41)	0.127
BMI, kg/m^2^	27.04 ± 5.6	28.2 ± 7.7	0.907.
BSA, m^2^	2.0 ± 0.2	2.0 ± 0.2	0.847
NYHA class, *n* (%)			
I	0	1 (5)	0.690.
II	6 (29)	10 (45)	0.569
III	12 (57)	13 (59)	0.754
IV	1 (5)	0	0.785
Systolic PAP, mmHg	47 ± 7	53 ± 7	0.124
RVEF, %	44.9 ± 12.6	55.1 ± 9.8	**0.008**
NT-proBNP, ng/l	1000 (557–2689)	450 (109–1000)	**0.007**
FVC, % predicted	72.6 ± 18.3	81.6 ± 14.9	0.217
Capillary CO_2_, mmHg	31 ± 4	35 ± 4	**0.001**
**Diaphragm ultrasound**			
Diaphragm excursion			
Amplitude during TB, cm	2.4 ± 2.3	2.5 ± 1.2	0.243
Velocity during TB, cm/s	1.7 ± 0.7	1.7 ± 1.0	0.836
Amplitude during VS, cm	2.8 ± 0.8	3.0 ± 1.1	0.498
Velocity during VS, cm/s	3.4 ± 1.0	5.7 ± 1.5	**<0.001**
Amplitude during maximal inspiration, cm	4.8 ± 1.3	5.2 ± 1.3	0.308
Diaphragm thickness			
FRC, cm	0.26 ± 0.08	0.20 ± 0.08	**0.013**
Thickening ratio	1.5 ± 0.3	2.0 ± 0.5	**0.001**

Values are mean ± standard deviation, median (interquartile range) or number of patients (%). Abbreviations: BSA, body surface area; Capillary CO_2_, capillary carbon dioxide level; NT-proBNP, N-terminal pro-brain natriuretic peptide; PCA, prostacyclin analog; PDE 5-I, phosphodiesterase 5 inhibitor. Significant *P*-values are represented in bold.

SniffV and DTR were weakly–moderately correlated with RVEF (SniffV: r = 0.34, *P*=0.03; DTR: r = 0.44, *P*=0.004) and pCO_2_ (SniffV: r = 0.42, *P*<0.001; DTR: r = 0.39, *P*<0.001).

### Predictors of exercise intolerance

**Figure 3:**
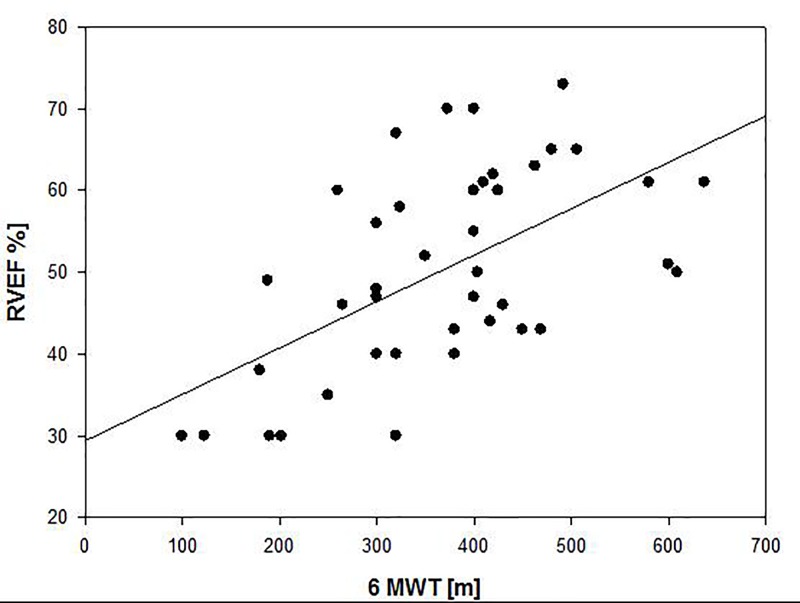
Correlation between RVEF and distance achieved in 6-min walking test (6MWD) in patients with precapillary PH

RVEF, NT-pro-BNP, capillary CO_2_, and both SniffV and DTR predicted 6MWD on univariate analysis (Table 4 and Figure 2). However, only RVEF and NT-pro-BNP remained independent predictors of 6MWD on multivariate analysis (Table 4 and Figure 3). Combining RVEF and NT-pro-BNP it was possible to explain 74% of the variability in 6MWD (Table 4).

**Table 4:** Predictors of 6MWD

	Univariate analysis	Multivariate analysis
**Hemodynamic variables**		
Systolic PAP	-	-
RVEF	r = 0.58; *P*<0.001	r = 0.47; *P*=0.001
NT-pro-BNP	r = −0.46; *P*=0.002	r = −0.27; *P*=0.047
Respiratory variables		
Capillary CO_2_	r = 0.52; *P*<0.001	-
FVC	-	-
**Diaphragm ultrasound**		
Amplitude during TB		
Velocity during TB	-	-
Amplitude during VS	-	-
Velocity during VS	r = 0.39; *P*=0.009	-
Amplitude during maximal inspiration	-	-
Thickness at FRC	-	-
Thickening ratio	r = 0.36; *P*=0.017	-

Abbreviations: Capillary CO_2_, capillary carbon dioxide level; NT-pro-BNP, amino-terminal pro-brain natriuretic peptide.

## Discussion

The present study confirmed that diaphragm impairment is a very common finding in patients with precapillary PH. Diaphragm dysfunction seems to develop as exercise intolerance progresses. However, the most important new finding of the present study is that inspiratory muscle strength impairment did not independently contribute to exercise intolerance in precapillary PH, beyond right ventricular systolic dysfunction.

Several factors predicting exercise intolerance in patients with precapillary PH have been found in previous studies [3–6]. However, these have not often been investigated with multivariate analysis. This is important because many pathophysiological determinants of exercise intolerance in patients with precapillary PH are likely to be interrelated. In fact, it is reasonable to assume that long-term increases in PAP and pulmonary vascular resistance would lead to RV dysfunction with reduced cardiac output, resulting in muscular hypoperfusion and myopathy, eventually causing hypertrophy and failure [5]. The myopathy might also involve the diaphragm, which has an increased metabolic demand due to chronic hyperventilation [3].

It is already known that assessment of RV systolic function is superior to invasive measurement of PAP in explaining exercise intolerance and even providing prognostic discrimination in precapillary PH [15–17]. For this reason it seems wise to also evaluate RV systolic function whenever testing a novel potential predictor of exercise intolerance in precapillary PH, such as diaphragm function.

Recently, inspiratory muscle strength has emerged as a new candidate explaining exercise intolerance in PH [8,9]. Indeed, lower values of inspiratory muscle strength as assessed by transdiaphragmatic pressure following magnetic stimulation of the phrenic nerve roots were shown to relate to exercise intolerance in precapillary PH patients [8]. Diaphragm ultrasound has emerged as an alternative, non-invasive, and more practical bedside tool to study inspiratory muscle function [10].

In the present study we clearly showed that ultrasound assessment of diaphragm function may identify compromised diaphragm strength in patients with precapillary PH. Indeed, there was almost no overlap in SniffV and DTR values between patients and controls. Only two previous studies investigated the contribution of inspiratory muscle dysfunction to exercise intolerance in precapillary PH [8,9]. While maximal inspiratory pressure measured through a mouthpiece did not predict exercise intolerance in patients with precapillary PH, [9] a subsequent study by Kabitz and coworkers [8] using magnetic phrenic nerve stimulation found that transdiaphragmatic pressure be decreased by 34% in patients with precapillary PH compared with controls and was related to exercise intolerance.

The magnitude of the reduction in diaphragmatic strength observed in precapillary PH patients in the present study (35% reduction in VS and 40% reduction in DTR versus healthy controls) and its correlation with exercise intolerance is close to that reported by Kabitz and coworkers [8] and supports the validity of ultrasound assessment of inspiratory muscle strength assessment in this clinical scenario. This makes diaphragm ultrasound a promising diagnostic tool for assessment of diaphragm function in patients with precapillary PH.

In precapillary PH, increased pressure in the pulmonary arteries overloads the RV, eventually causing hypertrophy and failure [5]. Most measures that predict survival in PH, such as exercise capacity and functional class, have been related to RV function [5]. In our study, RVEF determined using cardiac MRI (the gold standard) and natriuretic peptide hormone levels, were shown to be the only independent predictors of exercise intolerance; in fact, RVEF alone explained almost 50% of the variability in 6MWD. This suggests that RV systolic function is a stronger predictor of exercise tolerance than the other univariate predictors, such as pCO_2_ (which reflects baseline hyperventilation), and diaphragm strength (as reflected by SniffV and DTR, which have previously been reported to correlate with invasively obtained metrics of inspiratory muscle strength [10]). We hypothesize that RVEF may, on the one hand, cause hyperventilation through as yet undetermined reflex mechanisms (and thus a decrease in CO_2_) and is, on the other hand, also responsible for diaphragmatic impairment through chronic hypoperfusion.

Most of our pathophysiological understanding of diaphragm involvement stems from numerous studies on this topic in patients with systolic HF [18–21]. However, the impairment in inspiratory muscle strength in precapillary PH goes far beyond what has been reported in systolic HF (30–40 vs 10%) [22]. The higher degree of hyperventilation, as reflected by generally more severe hypocapnia in patients with precapillary PH versus HF, might be one explanation for this discrepancy [23,24]. Hyperventilation places higher demands on the inspiratory muscles, leading to more pronounced diaphragmatic dysfunction in the long run [23,24].

In light of the findings presented above (see Table 4) it may be speculated that diaphragm dysfunction does not play an important pathophysiological role in exercise intolerance in patients with precapillary PH. In particular the finding that robust markers reflecting right ventricular pump function (RVEF) and right ventricular overload (NT-pro-BNP) are the only independent predictors of 6MWD in the present study speaks out in favor of targeting right ventricular overload by lowering pulmonary pressure through vasoactive drugs as postulated in current guidelines [1]. This speculation would make diaphragm ultrasound appear rather unnecessary in this patient cohort either. However, diaphragm dysfunction may still be part of a vicious circle that underlies exercise intolerance in this patient cohort as detailed above. Therefore, further specifically designed and larger studies that use inspiratory muscle strength training protocols as a targeted approach are needed to further support the concept that an improvement in diaphragm function and inspiratory muscle strength helps improve exercise intolerance in this particular patient cohort. Of note, this has been shown to improve inspiratory muscle strength and functional capacity in 29 patients with PAH lately [25]. Therein diaphragm ultrasound may not only prove to be a valuable tool in clinical research but also (later on) potentially a tool that helps further monitor the impact and hence success of inspiratory muscle strength training in these patients.

In the current study, diaphragm ultrasound assessment was related to a volitional maneuver and is therefore subject to bias. However, the use of repeated measurements for each variable (mean over three samples) as well as the fact that the degree of diaphragm dysfunction was comparable with that observed using non-volitional assessment by magnetic phrenic nerve stimulation appear to support the validity of ultrasound in this setting. Plus, as per guideline recommendations, right heart catheterization was used to diagnose PH, but only echocardiography was repeated during follow-up visits, including at study enrollment. Although this may potentially introduce a lack of precision and exclude some relevant hemodynamic data, this topic has been evaluated in previous studies [5,15,17] and was not the focus of the current investigations. Likewise, it was not the primary focus of this study to assess and discuss diaphragm dysfunction in patients with precapillary PH due to chronic lung disease (chronic obstructive pulmonary disease in particular), as the pathophysiological background of it is likely substantially different from patients with Nizza class I/IV precapillary hypertension where an obstructive lung function impairment pattern is rarely seen as evidenced in our patients cohort. However, in order to prove the concept that diaphragm dysfunction as evidenced by ultrasound criteria does also occur in this patient cohort we identified 11 patients (age 69 ± 10, 6 men, FVC 75 ± 15% predicted, FEV_1_ 40 ± 15% predicted) with COPD in whom we collected diaphragm ultrasound data and in whom the presence of precapillary class III PH is very likely based upon echocardiographic measures (Pa Sys 45 ± 5 mmHg and simultaneous ascertainment that systolic – as defined as a LVEF above 55% – and diastolic – as defined as a normal E/A ratio and E/E′ and normal left atrial diameter-function are normal, hence making confounding with Nizza class II PH due to left heart disease unlikely). In these patients diaphragm abnormalities similar to those seen in patients with Nizza classes I and IV were found suggesting that diaphragm dysfunction does also occur there. This was evidenced by significantly lower amplitude during maximal inspiration of 5.4 ± 1.5 cm and a significant reduction in DTR of 2.2 ± 0.3: both *P*<0.05 against a control group matched for age, gender, and BMI).

## Conclusions

Patients with progressive precapillary PH are characterized by diaphragm dysfunction which may develop in response to chronic hyperventilation. However, diaphragm dysfunction does not independently predict exercise intolerance beyond what can be explained by RV systolic function. Additional research is needed to further elucidate the underlying pathophysiological mechanisms that lead to diaphragm dysfunction in PH.

## Perspectives

- This prospective study was conducted in patients with precapillary PH and age- and sex-matched controls to evaluate whether diaphragm function, assessed by diaphragm ultrasound, contributes to exercise intolerance, evaluated by the 6-min walking test, over and above impaired right ventricular pump function, as assessed by CMR imaging.
- The present study confirmed that diaphragm impairment is a very common finding in patients with precapillary PH. Diaphragm dysfunction seems to develop as exercise intolerance progresses. However, the most important new finding of the present study is that inspiratory muscle strength impairment did not independently contribute to exercise intolerance in precapillary PH, beyond RV systolic dysfunction.
- Patients with progressive precapillary PH are characterized not only be RV systolic dysfunction but also by diaphragm dysfunction which may develop in response to chronic hyperventilation. Additional research is needed to further elucidate the underlying pathophysiological mechanisms that lead to hyperventilation and diaphragm dysfunction in PH, since these alterations are not (yet) part of a targeted therapy in PH.

## Role of sponsors

The study funders had no role in study design, data collection and analysis, preparation of the manuscript, or the submission process.

## Supporting information

**Supplemental Videos S1:** 

**Supplemental Videos S2:** 

**Supplementary Material:** 

## Abbreviations

BMI: body mass index
CMR: cardiac magnetic resonance
CTEPH: chronic thromboembolic pulmonary hypertension
DTR: diaphragm thickening ratio
ECG: electrocardiogram
FEV_1_: forced expiratory volume after 1 s
FRC: functional residual capacity
FVC: forced vital capacity
IQR: interquartile ratio
PAH: pulmonary arterial hypertension
PAP: pulmonary artery pressure
PH: pulmonary hypertension
RV: right ventricle
RVEF: right ventricular systolic ejection fraction
SniffV: excursion velocity during a Sniff maneuver
TB: tidal breathing
TLC: total lung capacity
VS: voluntary sniff
6MWD: 6-min walk distance
