# Supplementary material for: Diaphragm function does not independently predict exercise intolerance in patients with precapillary pulmonary hypertension after adjustment for right ventricular function
Source: Biosci Rep. 2019 Sep 3;39(9):BSR20190392. doi: 10.1042/BSR20190392 (PMC6723707; doi:10.1042/BSR20190392)
Supplement: Supplementary file 3 [file bsr20190392_Supp3.pdf]

Abbreviation List

BMI:	body mass index f
CMR:	cardiac magnetic resonance
CTEPH:	chronic thromboembolic pulmonary hypertension
DTR:	diaphragm thickening ratio
FRC:	functional residual capacity
FVC:	forced vital capacity
HF:	heart failure
NT-proBNP:	amino-terminal pro-brain natriuretic peptide
NYHA:	New York Heart Association
PAH:	pulmonary arterial hypertension
PAP:	pulmonary artery pressure
PH:	pulmonary hypertension
RV:	right ventricle
RVEF:	right ventricular ejection fraction
6MWD:	6-minute walking distance
SniffV:	excursion velocity during a Sniff maneuver
TB:	tidal breathing
TLC:	total lung capacity
VS:	voluntary Sniff maneuver
